# Immune Fingerprint in Diabetes: Ocular Surface and Retinal Inflammation

**DOI:** 10.3390/ijms24129821

**Published:** 2023-06-06

**Authors:** Madania Amorim, Beatriz Martins, Rosa Fernandes

**Affiliations:** 1 Coimbra Institute for Clinical and Biomedical Research (iCBR), Faculty of Medicine, University of Coimbra, 3000-548 Coimbra, Portugal; 2 Institute of Pharmacology and Experimental Therapeutics, Faculty of Medicine, University of Coimbra, 3000-548 Coimbra, Portugal; 3 Center for Innovative Biomedicine and Biotechnology (CIBB), University of Coimbra, 3004-531 Coimbra, Portugal; 4 Clinical Academic Center of Coimbra (CACC), 3004-561 Coimbra, Portugal

**Keywords:** diabetes, diabetic retinopathy, ocular surface, retina, immunity, inflammation

## Abstract

Diabetes is a prevalent global health issue associated with significant morbidity and mortality. Diabetic retinopathy (DR) is a well-known inflammatory, neurovascular complication of diabetes and a leading cause of preventable blindness in developed countries among working-age adults. However, the ocular surface components of diabetic eyes are also at risk of damage due to uncontrolled diabetes, which is often overlooked. Inflammatory changes in the corneas of diabetic patients indicate that inflammation plays a significant role in diabetic complications, much like in DR. The eye’s immune privilege restricts immune and inflammatory responses, and the cornea and retina have a complex network of innate immune cells that maintain immune homeostasis. Nevertheless, low-grade inflammation in diabetes contributes to immune dysregulation. This article aims to provide an overview and discussion of how diabetes affects the ocular immune system’s main components, immune-competent cells, and inflammatory mediators. By understanding these effects, potential interventions and treatments may be developed to improve the ocular health of diabetic patients.

## 1. Introduction

Diabetes is a chronic disease caused either by insufficient insulin production by the pancreas or inefficient insulin use by the body. Globally, the estimated prevalence of this disease has more than tripled, from 151 million in 2000 to more than 537 million in 2021 [1]. If current trends persist, the predicted number of diabetics will rise to an astonishing 783 million by 2045. The main pathophysiological pathways of diabetes include hyperglycemia, dyslipidemia, insulin resistance, and metabolic dysregulation, which can seriously harm many body systems. With a thorough knowledge of diabetes, it is believed that oxidative stress, autoimmunity, genetics, and epigenetics play major roles in the onset of diabetes and its complications [2]. Although the most frequent and well-known ocular complication of diabetes is diabetic retinopathy (DR), with approximately one-third of all people with diabetes experiencing related vision complications, recent reports have focused on how diabetes affects the ocular surface [3,4], a structure similarly impacted by diabetes [5].

The human eye is a highly complex structure, with various components that work in well-orchestrated mechanisms making it an immune-privileged organ. The ocular surface is a complex structure outfitted with defenses against physical, chemical, infectious, and environmental harm. It is remarkable in how it controls immune function and acts as a barrier against pathogen invasion. However, the retina is also composed of a set of anatomic barriers and highly specialized cells that work synergistically to maintain retinal homeostasis and visual function [6]. (Intra)ocular inflammation caused by an infection or autoimmunity in Type 1 diabetes plays a key role in several diseases impairing vision, including DR [7,8,9]. DR is a chronic disease that progresses through increasing severity stages [10]. In individuals with type 1 diabetes, nearly 95–97% develop retinopathy within a span of 20 years [11,12]. At the time of initial diabetes diagnosis, approximately 21% of patients with type 2 diabetes already have DR; over time, more than 60% develop some degree of retinopathy [1,13]. It is important to note that the progression of DR varies among individuals and is influenced by the dynamic nature of the disease’s natural history [14]. Interestingly, some patients develop DR without exhibiting the traditional risk factors typically associated with the condition, such as the long duration of diabetes, poor blood glucose and blood pressure control, and dyslipidemia. Conversely, there are individuals with long-standing diabetes and inadequate glycemic control who do not develop DR. These observations highlight the complexity of the disease and emphasize the need for a deeper understanding of its underlying mechanisms. The pathophysiology of DR is complex since hyperglycemia induces multiple pathological changes in the retina´s neuronal and vascular components, which reflects the interconnected nature of all the cellular components in the tissue. Moreover, local and/or systemic inflammation linked to diabetes affects virtually all ocular cells, leading to multi-cellular dysfunction, dysregulated physiological responses, and organ failure [14]. A growing body of evidence has shown that the immune system, composed of immune-competent cells, inflammatory mediators, and the complement system, plays a key role in DR progression and may be the underlying cause of homeostatic dysregulation in all stages of the disease [15].

In this review, we summarize the current understanding of the impact of diabetes on the ocular surface and retina and the underlying molecular and cellular dysregulation of innate immunity. We further discuss the challenges of translating the knowledge obtained from findings using cellular and animal models of diabetes to human disease.

## 2. Ocular Surface Environment in Health

**Figure 1:**
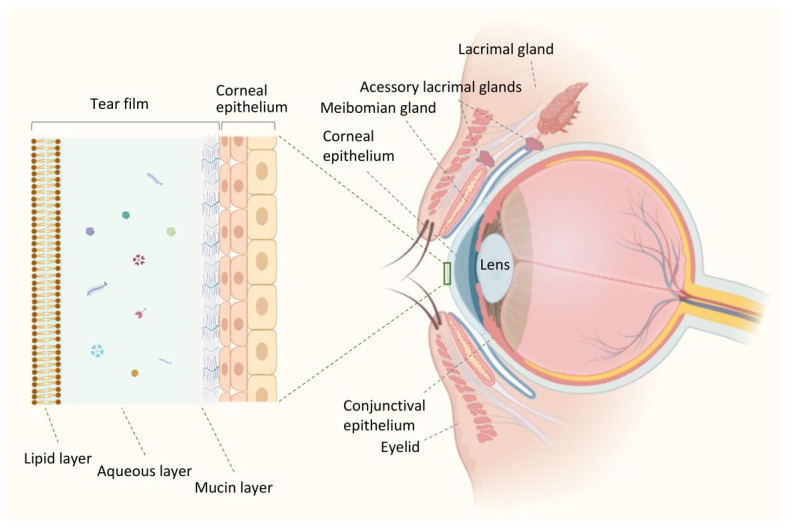
Components of the ocular surface and the three-layered structure of the tear film. The ocular surface comprises the cornea and conjunctival epithelium, as well as the main and accessory lacrimal glands, the Meibomian gland, and the eyelid structures. Together, they form a single and integrated functional unit essential for maintaining the eye’s health. The tear film, which covers the ocular surface, is composed of three layers. The inner mucin layer is made up of secreted and membrane-bound mucins that allow the tear film to adhere to the corneal epithelium by reducing the hydrophobicity of the epithelial cells. The intermediate aqueous layer, consisting of water, electrolytes, immunoglobins, glycoproteins, antimicrobial peptides, cytokines, vitamins, and hormones, is derived from the main and accessory lacrimal glands and is primarily composed of water, which provides a smooth refracting surface. The outer superficial lipid layer, secreted by Meibomian glands, helps preserve the tear film’s stability and integrity. This illustration was created using BioRender.com, accessed on 4 May 2023.

The ocular surface comprises the surface and epithelia of the cornea, conjunctiva, accessory lacrimal gland, meibomian gland, and eyelid structures (Figure 1). The lacrimal apparatus and the interconnecting innervation (sensory and motor nerves) constitute the lacrimal functional unit (LFU). LFU is formed by distinct components that work in an integrated manner to regulate the volume and composition of the tear film through the activation of corneal sensory inputs, which are processed by the secretory apparatus, thus contributing to maintaining the ocular surface health [16,17].

The surface of the eye exposed to the environment, and the internal compartments (anterior and posterior segments of the eye) contain many soluble immunoregulatory and immunosuppressive molecules that regulate apoptosis, promote the production of anti-inflammatory mediators, and influence the activity of immune cells [18]. The external surface of the eye, composed of mucosal tissue (conjunctiva and cornea), is constantly exposed to external threats. The cornea is an optically transparent tissue of the anterior part of the eye that functions as a chemical and mechanical barrier, preventing the penetration of foreign bodies [18]. Protection of the ocular surface and integrity of the barrier is due mainly to the presence of substances with an antibacterial function present in the tear film and produced by immune cells and cells at the ocular surface [19]. Any perturbations by environmental factors and/or pathogens can cause inflammation or ocular infection, disrupting immune system homeostasis at the ocular surface and thus threatening vision.

Essential functions of the ocular surface system include protecting, nourishing, and ensuring a smooth refractive surface on the cornea. Another function is related to the maintenance of continuous corneal and conjunctival epithelia with no breaks and the communication along these epithelia through gap junctions and cytokines [20,21,22]. In addition, all cells at the ocular surface produce the tear film components [16]. The continuity of epithelia, innervation, endocrine, circulatory, and immune systems functionally link all the constituents of the ocular system. They are supported by connective tissue with its resident cells and blood vessels [16]. Signals from one region influence the blink, goblet cell secretion, lacrimation, and/or lacrimal gland gene expression, making the ocular surface a closed integrated system [16].

The corneal and conjunctiva epithelia, and their cell–cell junctions, serve as crucial anatomical barriers in the eye, protecting it from external threats [17]. The apical cell membranes on superficial cells provide an additional layer of protection [23], composed of a hydrophilic, heavily glycosylated glycocalyx [16] consisting of transmembrane mucins (MUC), which are carbohydrate–protein complexes of high molecular weight glycoproteins with O-linked carbohydrates and a protein core [24]. These mucins associate with carbohydrate-binding proteins, such as galectin-3, which interacts with carbohydrate residues on MUC1 and MUC16, contributing to the integrity of the epithelial barrier [23]. Mucins also play a role in maintaining epithelial surface hydration and lubrication. Apart from epithelial cells, fibroblasts and Langerhans cells are essential components of the corneal innate immune system [23].

Moreover, the cornea has the highest density of sensory nerves in the body, ranging from 300 to 600 times greater than the skin [25]. As a result, it exhibits exceptional sensitivity. It can effectively respond to harmful physical and chemical stimuli, although the number of sensory fibers detecting innocuous environmental changes is comparatively small [26].

The tear film is a complex and thin fluid, about 2–5.5 mm thick, that serves several crucial functions as the interface between the ocular surface and the surrounding environment [27]. It provides an optically smooth surface, lubricates, and protects the cornea and eyelid interface from the external environment and immunological factors [28].

Traditionally, a three-layered model of the tear film was used to represent in a simple way the tear film [29], which consists of (a) an inner mucin layer covering the ocular surface and lowering the hydrophobicity of the corneal epithelial cells; (b) a middle aqueous layer forming the largest part of the tear film thickness and providing nutrients; (c) and an outer lipid layer preventing evaporation of tears (Figure 1). Although this is a well-established representation of the tear film, fresh viewpoints that might improve our comprehension of the dynamics, structure, and function and the alterations that result in pathological states can be limited by this model with three separate and distinct layers. It is now believed that a mucin gradient is created in the aqueous layer, mixing these two layers to form a hydrated gel covered by the lipid layer. In this way, the tear film behaves as a single dynamic functional unit [24].

The mucous layer is mainly secreted by the conjunctival goblet cells, corneal and conjunctiva epithelium, and the lacrimal gland [24]. It comprises mucins that form the glycocalyx covering the mucosal epithelial cells, allowing the tear film to adhere to the eye [30,31]. The mucous layer contributes to the stability of the tear film and helps ensure that the aqueous layer is constantly distributed over the ocular surface. Thus, the mucins in the tear film maintain the ocular surface hydrated, provide lubrication and prevent friction of the ocular surface against the conjunctiva during the blink of the eye. Additionally, they form a protective physical barrier preventing microbes’ adhesion and invasion [24]. Both corneal and conjunctival epithelial cells express membrane-bound mucins, such as MUC1, MUC2, and MUC4, while the conjunctival goblet cells secrete mucin MUC5AC [30]. Mucin production can also be induced by the inflammatory cytokines and the stimulation of Toll-like receptors (TLR) in the corneal epithelial cells [30]. The aqueous layer constitutes more than 90% of the total tear film thickness and is secreted by the principal and accessory lacrimal glands [24]. This layer comprises basically 98% water, organic components such as proteins/peptides (enzymes, growth factors, cytokines, antimicrobial peptides (AMPs), small molecule metabolites, and inorganic electrolytes [24,31,32,33]. Corneal and conjunctival cells also secrete proteins/peptides and small molecule metabolites into the tear film [24].

The primary function of this layer is to form a smooth refractive surface together with the corneal epithelium [31]. The presence of electrolytes, mainly sodium, potassium, chloride, bicarbonate, and also magnesium and calcium in lower levels in the aqueous layer contribute to the maintenance of the osmolality of the tear film (300–305 mOsm/kg in healthy eyes), which represents a measurement for the balance between tear production, evaporation, drainage, and absorption [34]. In addition to that, electrolytes within the aqueous layer play a critical role in maintaining epithelial integrity and regulating the tear fluid’s pH [34,35]. The lipid layer forms the outermost layer of tear fluid and is approximately 42 nm thick [36]. The meibomian, Moll, and Zeis glands secrete lipids [24]. The lipid layer mainly comprises cholesterol, ester, and ether lipids, whose primary function is to prevent rapid evaporation of the tear film’s aqueous layer [24,31,32,33]. So, the lipid layer is crucial for preserving the stability and integrity of the tear film. It may also provide a barrier against pathogens [24]. A study by Green-Church et al. (2011) has proposed a model of the precorneal tear film, in which the lipid layer is further divided into two sublayers, the outer non-polar lipid layer and the inner polar lipid layer with intercalated proteins [37].

Analysis of the human tear proteome has revealed that it is a complex biological fluid containing approximately 1500 proteins [24]. The proteins are secreted from the lacrimal gland, ductal epithelium, and associated plasma cells [33]. Proteins such as lactoferrin, lysozyme, secretory immunoglobulin A (sIgA), lipocalin, superoxide dismutase (SOD), cystatins, and alpha-1 protease inhibitors are highly present, comprise more than 90% of the total amount of tear proteins. The remainder of protein components includes growth factors, neurotrophic factors, cytokines, cell adhesion, molecules, matrix metalloproteinases (MMPs), immunoglobulins, and insulin [33].

Several lysosomal enzymes (acid hydrolases) are present in tears in concentrations 2–10 times higher than the serum. The lacrimal gland is the primary source of the lysosomal enzyme. Two of the more abundant proteins of the tear film are lysozyme, an antibacterial enzyme, and lactoferrin, an iron-binding protein whose function is to inhibit bacterial growth. Lipocalin binds and transports small hydrophobic molecules, acting as a lipid scavenger from the corneal surface. The secretory immunoglobulin A (sIgA) is the primary antibody in tears and is crucial in the host ocular immune response [33].

Lipidomic analysis of secretion from meibomian glands revealed that almost all lipid classes are represented, mainly hydrocarbons, wax esters, cholesterol esters, triglycerides, and in lesser amounts, diglycerides, monoglycerides, free fatty acids, free cholesterol, and phospholipid [38,39]. Moreover, it has been shown that the lipid profiles in tears and meibomian glands are nearly identical [39]. There is growing evidence that the typical tear film contains several pro- and anti-inflammatory cytokines. These molecules are secreted not only by ocular surface epithelial cells but also by immune system cells. Although the secretion of cytokines and chemokines by epithelial cells is usually increased upon cell stimulation, in basal (unstimulated) tears, secretion of those mediators also occurs [40].

## 3. Ocular Surface Innate Immunity

Along with environmental influences, innate immunity oversees nonspecific initial host protection mechanisms against infections. Additionally, it contributes to controlling inflammation, preserving immunologic homeostasis, and activating adaptive immunity [40]. The innate immune system, composed of host elements and local microbiome (commensal resident microorganisms) [41], is the first mechanism for host defense, promoting the production of both proinflammatory and anti-inflammatory mediators. Different components make part of the innate system: (i) physical barriers created by tight junctions in epithelial and mucous membrane surfaces; (ii) inflammation-related serum proteins, such as complement proteins, lectins, ficolins, and C-reactive protein; (iii) antimicrobial peptides (AMPs), such as cathelicidin defensins, among others; (iv) phagocytes; (v) pattern recognition receptors (PRRs) such as TLR; (vi) cells that secrete inflammatory mediators, such as macrophages, natural killer, and dendritic cells [41].

Antimicrobial peptides (AMPs), often referred to as host defense peptides (HDP) [41,42], are a category of secreted PRRs released by epithelial cells in their injured sites, counteracting the invasion and colonization of pathogens at the ocular surface [43]. AMPs are a diverse class of molecules with powerful immunomodulatory and antibacterial properties and are less than 100 amino acids in length and coded by separate genes [44,45]. Defensins, cathelicidins, and S100A proteins are the three main classes of AMPs found in humans. Based on their secondary structure, AMPs are divided into four main classes: alpha helices, beta strands, loop structures, and extended structures [46]. The most prevalent classes in nature are the alpha-helix and beta-sheet [47]. Defensins found in mammals are beta-sheet peptides. AMPs usually have a positive net charge due to the high amount of cationic amino acids, and their structure has amphiphilic (both hydrophilic and hydrophobic) characteristics. Electrostatic interactions between these positively charged AMPs and the negatively charged phospholipids in the cell membranes of microorganisms result in membrane adsorption and conformational changes, and the hydrophobic sides are important to anchor in the hydrophobic lipid core of the bilayer [43,44,46]. These peptides’ hydrophobicity and number of cationic net charges correlate with the antibacterial and hemolytic activities, respectively [47]. In addition to their antimicrobial activity, AMPs have functions regarding proliferation, migration, chemotaxis, and cytokine production, therefore linking AMPs with the adaptive immune system [44,46].

Several studies have shown that ocular surface cells express several AMPs, some constitutively, and others are induced by infection, inflammation, and in response to microorganisms [46]. α-defensins (defensins 1, 2, and 3 produced by the resident or non-resident neutrophils and possibly by secretion from lacrimal ductular epithelium), β-defensins (defensin 1 and 2 produced by the ocular surface) and human cathelicidin LL-37 are the primary AMPs present at the ocular surface [44,45]. Human neutrophil peptide (HNP) 1–3, MUC7, histatin, surfactant protein D (SPD), liver13 expressed antimicrobial peptides (LEAP)-1 and -2, macrophage inflammatory protein (MIP)-3, DEFB109, and RNase-7 are other AMPs less present [42,43]. These AMPs have been detected in cells from the lacrimal gland, Krause and Moll glands, cornea, and conjunctiva epithelia [44]. Defensins have a wide range of antimicrobial activity against Gram-positive and Gram-negative bacteria, fungi, and viruses, creating voltage-sensitive channels in the plasma membrane of the microorganism. They also promote a quick cellular immune response to an infection via a chemotactic effect and accelerate wound healing by the mitogenic effect on epithelial cells and fibroblast [48]. Human beta-defensin 1 (hBD1) is constitutively expressed, whereas human beta-defensin 2 (hBD2) is inducible by exposure to lipopolysaccharide and peptidoglycan from bacteria, proinflammatory cytokines and in response to injury [43].

The cathelicidin, LL-37, is naturally present in the conjunctiva and cornea, and its expression increases in response to corneal injury, infections, and other inflammatory processes [49]. Its functions include the recruitment of inflammatory cells, wound healing, promotion of angiogenesis, proprieties as a chemotactic agent for neutrophils, monocytes, and T cells, regulation of cell death pathways, and antimicrobial activity [45,50].

Through PRRs, the innate immune system recognizes pathogen-associated molecular patterns and destroys them through phagocytosis, cytokine release, natural killer cells, and complement system [51].

The corneal’ s innate immune system comprises many types of cells, including epithelial cells, fibroblasts, macrophages, and dendritic cells. Epithelial cells are responsible for secreting cytokines to activate the immune system against microbial invasion [17,52]. Those cytokines are TNF-α, IL-1, IL-6 and IL-8 [17]. When the cell membrane of epithelial cells is ruptured by infectious agents or trauma, interleukin (IL)-1α, which is stored in them, is passively released [53]. Chronic IL-1α secretion would lead to cornea destruction due to amplified immune invasion and neovascularization [52], so cornea epithelial cells can modulate IL-1α secretion. It is postulated that they can secrete the soluble and membrane-bound forms of the IL-1α receptor (IL-1RII), a natural IL-1α antagonist [54].

Fibroblasts in the stroma may be in charge of the production of IL-1, IL-6, IL-8, TNF-α, and α-defensin as a way of after microbial infections [17]. Dendritic cells (DCs) are antigen-presenting cells. It is currently known that different subtypes reside in the cornea, distributed in a peculiar way, where their number reduces from the periphery to the center of the cornea [55,56,57,58]. DCs can be subdivided into three main groups: the conventional DCs (cDCs), the plasmacytoid DCs (pDCs), and the monocyte-derived DCs (moDCs) [59]. Langerhans Cells (LCs), a subtype of conventional DCs (cDC), are observed in the periphery and center of the cornea [60,61,62,63,64]. The interstitial DCs, another subtype of conventional DCs, are located in peripheral, paracentral, and central regions of the anterior stroma in the cornea [60,61,64,65]. Plasmacytoid dendritic cells (pDCs) have also been observed in the anterior stroma and the central and peripheral cornea epithelium [64]. Cornea DCs play an immunological role in inducing and amplifying immunoinflammatory responses [66,67]. During inflammatory processes, they are activated through the release of pro-inflammatory cytokines [56,57]. On the other hand, DCs are associated with non-immunological functions such as tissue repair. Upon injury, corneal DCs are activated either directly through recognition of injury signals or indirectly from cytokines and chemokines secreted by epithelial cells in the injury site. Once activated, they modulate the migration, proliferation, and survival of epithelial cells in the wounding area through cell-to-cell contact or the release of survival and growth factors [68].

Corneal epithelial cells, dendritic cells, and cornea sensory neurons are anatomically and structurally intimately connected, a concept of epithelium–nerve–dendritic cells (epineuroimmune) function unit, which is analogous to the neurovascular function unit in the retina, which consists of glia, vasculature, and neurons, has been proposed [48]. So far, little is known about the physiological mechanisms of intercommunication between the components of this unit, to the point of questioning which of them initially detects the injuries or alterations and induces changes in the other two components [69]. The microbiota of the ocular surface is composed of microorganisms that colonize the cornea and conjunctiva [70]. A diverse microbial community in the healthy conjunctiva has been reported, with 12 ubiquitous core genera. *Pseudomonas*, *Propionibacterium*, *Bradyrhizobium*, *Corynebacterium*, and *Acinetobacter* were the most abundant. *Brevundimonas*, *Staphylococci*, *Aquabacterium*, *Sphingomonas*, *Streptococcus*, *Streptophyta*, and *Methylobacterium* were also present but accounted for a low percentage of the putative core of the ocular surface microbiome [71]. However, many ocular pathogens belong to these core genera at the ocular surface, namely *Pseudomonas*, *Acinetobacter*, *Propionibacterium*, *Corynebacterium*, *Staphylococcus*, *Streptococcus*, and *Sphingomonas.* The presence of diverse potentially pathogenic bacteria on the healthy ocular surface suggests that powerful mechanisms may exist at the ocular surface in suppressing microbial pathogenicity [71]. According to previous studies, these could include interactions with the immune system and commensal bacteria [72]. Locally present opportunistic microorganisms may bring some eye infections after external conditions have increased their aggressiveness rather than by foreign invaders. In other human settings, it has been found that aging, trauma, and the reduction of the local commensal microbiota cause a rise in virulence [73,74].

## 4. Homeostasis of the Innate Immune Network in the Retina

The retina, a central nervous system component, exhibits a tight coupling between neuronal activity and local blood flow. An adaptive vascular network helps supply more oxygen and nutrients to meet the increased metabolic needs of active neurons. Due to its high metabolic demand and constrained vascular supply, the retina is acutely sensitive to changes in metabolism [75,76,77]. Importantly, the innate immune system preserves the environment’s homeostasis and visual function by acting as the first line of defense against environmental disturbances [6]. The innate immune system regulates and maintains a large portion of this homeostasis in a set of critically important anatomic barriers made up of and controlled by retinal cell types with a high degree of specialization.

**Figure 2:**
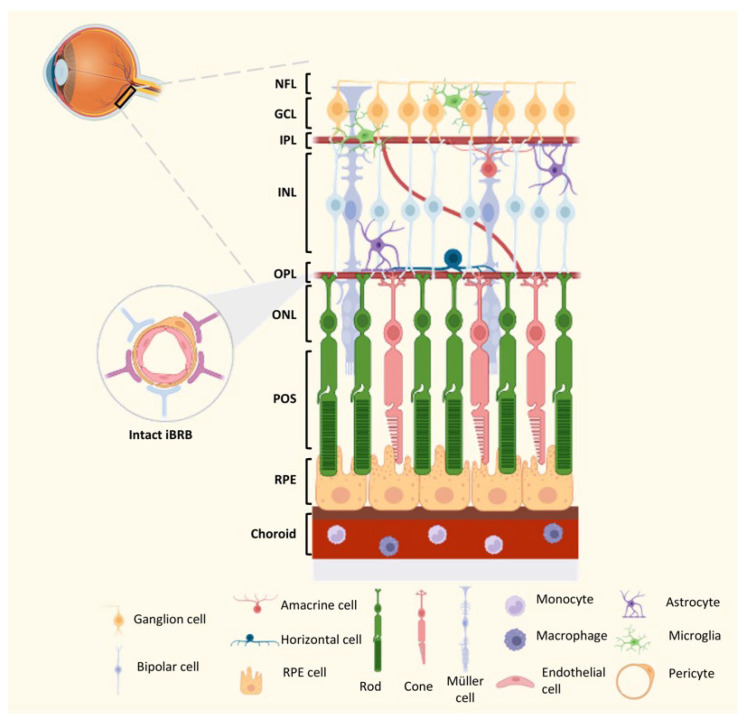
Schematic representation of the homeostatic immune environment of the retina. Under homeostatic conditions, the resident microglia and the astrocytes mainly occupy the ganglion cell layer (GCL) and both plexiform layers. Along with Müller cells, which span all the retinal layers, and the main components of the inner blood–retinal barrier (iBRB), such as endothelial cells and pericytes, they form the neurovascular unit (NVU), which help maintain the immune privilege and overall health of the retinal. NFL—Nerve fiber layer; GCL—Ganglion cell layer; IPL—Inner plexiform layer; INL—Inner nuclear layer; OPL—Outer plexiform layer; ONL—Outer nuclear layer; POS—Photoreceptor outer segments; RPE cell—Retinal pigment epithelial cell. This schematic illustration was created using BioRender.com, accessed on 4 May 2023.

The inner and outer blood–retinal barriers (BRB) are anatomic barriers that prevent entry into the retina of circulating immune and inflammatory cells, being dynamic barricades for maintaining the immune privilege of the ocular environment. The former is a neurovascular unit (NVU) comprised of endothelial cells, pericytes, and glial (astrocytes, microglia, and Muller) cells that work together to ensure visual function and eye health (Figure 2) [15]. This barrier prevents molecules, and circulating cells from reaching the delicate neuroretinal tissue from systemic circulation. Like the inner BRB, the latter is also formed by tight junctions that join the epithelial cells of the retinal pigment epithelium. In addition to acting as a barrier, preventing the entry of harmful substances or pathogens from the bloodstream into the retina, RPE cells have immune-modulatory functions, including the secretion of immune-regulatory molecules that help maintain retinal immune privilege and prevent inflammatory responses. Thus, in addition to limiting the retina´s exposure to external elements, both barriers control the retina´s access to nutrients from the vasculature [15].

In addition to its physical barriers, the retina boasts a sophisticated immune defense system comprised of various types of glial cells (such as microglia, astrocytes, and Müller cells), perivascular macrophages, and the complement system (Figure 2). Together, these components act as a formidable protective shield against potential threats. Microglia cells, one of the cell types of the NVU, are innate immune cells of myeloid origin that play a vital role in maintaining retinal homeostasis and are involved in various functions, including microenvironment surveillance, by extending and retracting their processes [78,79], clearance of degenerating cells by phagocytosis [80,81], and participation in inflammation through the secretion of cytokines and growth factors [81]. These immune cells are crucial in surveilling the retina, patrolling for any signs of infection or tissue damage, and promptly responding to potential threats. Due to their strategic location at the interface between the systemic circulation and the neuroretina, microglia establish an intricate network of relationships with macroglial cells, which is essential for their functions. Müller cells constitute the main source of adenosine triphosphate, which fuels microglia´s energy-intensive activities [82]. Astrocytes, another subtype of glial cells in the central nervous system, are highly plastic cells and influence the tightness of the BRB by secreting factor factors [83]. Other innate immune cells, such as hyalocytes, perivascular macrophages, and dendritic cells, have been proposed to regulate the peculiar retinal immunological state (Figure 2) [15].

Furthermore, the retina has a unique and tightly regulated immune environment called immune privilege, enabling it to endure foreign antigens without provoking an inflammatory response. This distinctive trait is attributed to the existence of specialized mechanisms that control immune cell activation and suppress immune responses within the retina.

## 5. The Impact of Diabetes and DR on Ocular Surface and Tear Protein Profile

Diabetes can lead to various eye complications, but the most extensively studied and concerning of these is DR. Clinicians have been primarily focused on this condition due to its potential to cause severe vision impairment. Consequently, our current understanding of ocular complications associated with diabetes is centered mainly on the retina. However, it is now known that diabetes also has an impact on the ocular surface [5], leading to impaired function of the epineuroimmune function unit. Chronic hyperglycemia can significantly impact various ocular surface components, including the corneal epithelium, corneal nerves, tear film, endothelium, and conjunctiva [84]. Abnormalities in the cornea (delayed epithelial wound healing, edema, corneal erosions, neuropathy, decreased sensitivity) and tear film changes have been reported [85,86,87,88,89,90,91,92,93]. A healthy cornea depends on the appropriate functioning and communication of epithelial cells, sensory neurons, and resident immune cells, and diabetes disrupts their interaction and cooperation. While corneal problems are often overlooked, they are prevalent in patients with diabetes, affecting up to 70% of those examined [5]. Research suggests that impaired corneal epithelial wound healing and changes in immune cells in diabetic corneas may indicate more widespread disease. This underscores the importance of developing new screening tools to facilitate the early treatment of DR.

The cornea undergoes constant wear and tear and requires continuous regeneration. Therefore, any process that affects wound healing or the speed of corneal epithelium regeneration may have significant physiological implications and negatively impact one´s quality of life [94]. Studies have reported that diabetes is associated with decreased corneal oxygen consumption, abnormal collagen formation, altered glycosaminoglycan metabolism, and thickening of the corneal basement membrane [95]. Both type 1 and type 2 diabetes have been associated with reduced corneal nerve density and other abnormalities in corneal nerves. The reduction in corneal sensitivity, a characteristic manifestation of diabetic corneal neuropathy, has been proposed to result from demyelination of the nerves due to abnormal lipid metabolism and sorbitol accumulation within the Schwann cells [96]. Several cross-sectional clinic-based studies have reported that individuals with type 1 and type 2 diabetes have higher hysteresis, which suggests a more rigid and less deformable cornea [97,98,99,100,101]. More recent studies have also found that diabetes is associated with increased corneal thickness [94,102,103,104].

The exact reason why diabetes is associated with increased corneal hysteresis or thickness is not fully understood. However, it has been speculated that the accumulation of advanced glycation end-products (AGEs) in the corneal stroma of diabetics may result in non-enzymatic crosslinking between collagen molecules and proteoglycans [94]. Another complication associated with diabetes is epithelial keratopathy, characterized by delayed epithelial healing, increased endothelial permeability, and fragility, striate keratitis, accumulation of glycogen and glucose, microcystic edema, and bleb formation. Subconjunctival hemorrhages and/or microaneurysms are also common in diabetic patients [105].

In addition to ocular surface issues, diabetes also affects tear film homeostasis [106]. Studies have reported that diabetic patients have decreased tear secretion and impaired tear film function, which worsens with the progression of DR [107]. Altered tear film quality and quantity are observed in diabetic patients [108], leading to dry eye disease, which is very common among people with diabetes [106]. Alterations in tear film homeostasis appear to be negatively correlated with diabetes duration and have also been shown to correlate with the severity of DR.

Several factors related to diabetes contribute to the impact on tear film dynamics. It has been reported that diabetes impairs tear film production and function due to damage to the microvasculature of the lacrimal gland and autonomic neuropathy abnormalities [106]. Abnormalities in innervation can lead to alterations in tear production and impairment of motor and vegetative stimulus, resulting in neurotrophic lesions related to the tear dynamics and ocular surface homeostasis [109]. On the other hand, damage in corneal innervation interrupts the anti-inflammatory neural feedback [110]. It has been found to correlate with a reduction in goblet cells and mucin proteins [111,112], leading to alterations in tear film composition, stability, and functions. Studies have reported that long-term diabetes and poor blood glucose control can impair meibomian gland function, possibly due to insulin resistance/deficiency and hyperglycemia being deleterious to meibomian gland epithelial cells, as they impact the steroids and lipid receptors in the glands [109]. Insulin supports the metabolism and growth of the lacrimal gland, playing an important role in metabolic and mitogenic effects through the mediation of nutrient influx, energy storage, gene expression, and protein synthesis [113]. Therefore, either individually or combined, peripheral nervous lesions, glucometabolic disorder with chronic hyperglycemia, and impairment in insulin action may create an inflammatory environment [113], impairing tear film homeostasis in diabetic patients and leading to alterations in tear film secretion, function, and protein profile.

In the context of DR, several studies have reported changes in tear proteins of diabetic patients. For example, Herber et al. demonstrated a significant increase in tear film proteomics, while Yu et al. showed increased levels of secretory immunoglobulin A (sIgA) in diabetic patients and increased levels of lysozyme and decreased levels of lipocalin in patients with DR compared with healthy controls [107,114,115]. They also reported decreased levels of lactoferrin and lipocalin in patients with proliferative diabetic retinopathy (PDR), suggesting a decreased tear film function [115]. Kawai et al. reported increased apolipoprotein A-1 levels in patients with diabetes and DR [115]. Recently, we conducted a proteomic analysis of human tear fluid and reliably quantified 682 proteins. Among these, 32 proteins were found to be differentially expressed when comparing nondiabetic individuals, diabetic patients with no DR, and diabetic patients with nonproliferative DR (NPDR) or with PDR [85]. It is known that in diabetes and its complications, including DR, tear proteins can undergo glycation (non-enzymatic glycosylation) [116], resulting in the production of AGE-modified proteins that may impair protein function [108]. Zhao et al. reported that lactoferrin is particularly susceptible to glycation [108], and Moschos et al. reported increased levels of glycosaminoglycans in diabetic patients [115].

Tears, a complex fluid, have been found to change cytokine levels due to diabetes and DR. In a study conducted on patients with and without DR, it was observed that chronic inflammatory and angiogenesis processes might occur at the ocular surface and tear film, which can be reflected in changes in cytokine levels [117]. Tear samples from patients with DR showed increased ratios of IFN-γ/IL-5 and IL-2/IL-5 and decreased ratios of IFN-γ/IL-8, IL-4/IL-8, and IL-12p70/IL-8. On the other hand, diabetic patients with and without DR had elevated pro-inflammatory cytokines MCP-1 and IP-10, along with a compensatory increase in anti-inflammatory cytokine IL-1ra and decreased ratios of IFN-γ/MCP and IL-4/MCP-1 [118]. We found increased concentrations of IL-2/-5/-18, TNF, and MMP-2/-3/-9 compared to the control individuals [85]. Our group has reported higher concentrations of IL-2/-5/-18, TNF, and MMP-2/-3/-9 in tear fluid of type 2 diabetes with NPDR than in the control group [85]. Moreover, the concentrations of IL-5/-18 and MMP-3/-9 in tears were higher in the PDR group than in the controls [85].

Additionally, soluble TNF1 and two receptors correlate with the severity of DR [119]. Nerve growth factor (NGF) levels were found to be higher in tears of patients with DR compared to non-diabetic patients [120], and lipocalin-1, lactotransferrin, lysozyme C, lacritin, lipophilin A, and immunoglobulin lambda chain were identified as potential biomarkers in tears from patients with DR [121]. Furthermore, altered levels of LCN-1, HSP 27, and B2M were observed in the tears of diabetic patients compared to healthy controls, as reported by Kim et al. [122].

A recent study of conjunctival swabs using conventional culture and next-generation sequencing (NGS) analysis has reported that potentially pathogenic bacteria such as Enterobacteriaceae, Neisseriaceae, *Escherichia*–*Shigella*, and *Pseudomonas* were more prevalent in diabetes, particularly in DR. Dissimilarities in the ocular surface microbiome were also observed between diabetic and non-diabetic groups. Interestingly, the ocular surface microbiome in poorly controlled diabetic patients differs significantly from well-controlled diabetic and non-diabetic groups [3]. In poorly controlled diabetic patients, an inappropriate immune response can cause ocular surface microbiome dysbiosis and increase the abundance of potentially harmful bacteria, especially in DR [123,124,125,126].

## 6. Evidence of Changes in Ocular Surface and Immune Response in Experimental Models of Diabetes

Although the corneas of rodents and humans differ structurally, research has revealed comparable effects of chronic hyperglycemia on the ocular surface of diabetic rodents [127]. Several animal models of diabetes of type 1 diabetes and type 2 diabetes (C57BL/6J db/db mice, Sprague–Dawley rats in which diabetes was induced by injection with streptozotocin and spontaneously diabetic Goto–Kakizaki rats) have been used to mimic human corneal changes in diabetes. In vitro experiments showed that high glucose exposure harms human meibomian gland epithelial cells, altering their morphology and inducing cell death. It also leads to a reduction of IGF-1R, phospho(p)-AKT, Forkhead box O1 (FOXO1), and sterol-regulatory element binding protein (SREBP-1). Insulin exerted a dose-dependent increase in AKT signaling, mediated through IGF-R1, supporting the hypothesis that insulin resistance/deficiency may contribute to meibomian gland dysfunction [128]. In a diabetic animal model, pathological changes in meibomian glands (shrunk acini, destruction of acini basement membrane, disorganization of meibomian gland structure, and dropout of meibomian gland) were described four months after induction of diabetes with STZ [129]. In that study, increased inflammatory cell infiltration and elevated expression of inflammatory mediators (IL-1α, IL-1β, ELAM1, ICAM-1, and VCAM-1) were observed in diabetic meibomian glands. This inflammatory response seemed to be mediated through the activation of ERK1/2 and NF-kB p65 [129].

Decreased tear secretion, less innervation, delayed epithelial wound healing, and weakened cell junction was found in diabetic rats [130,131]. In diabetic mice in which scopolamine hydrobromide was systemically administered to induce dry eye, tear production significantly declined and displayed corneal epithelial defects [132]. Analysis of the cDNA array in healing epithelia of diabetic rodent corneas revealed significantly wounding-induced expression of the pro-inflammatory cytokine IL-1β [133] by endogenous danger signals or alarmins and stimulation of TLR [134]. Moreover, diabetes impaired PI3K-AKT signaling, induced apoptosis, diminished cell proliferation, repressed neutrophil and natural killer (NK) cell infiltration, and hampered sensory nerve re-innervation in healing mouse corneas. This study, using cytokine protein array, revealed the involvement of C-X-C motif chemokine ligand 5 (CXCL5; a chemokine that recruits and activates neutrophils), chemokine ligand (CCL)5 (a chemoattractant for monocytes and eosinophils) and CXCL10 (a chemoattractant that recruits and activates NK cells, acting as a downstream modulator of IL-1b-IL-1Ra-mediated signaling) [134].

**Figure 3:**
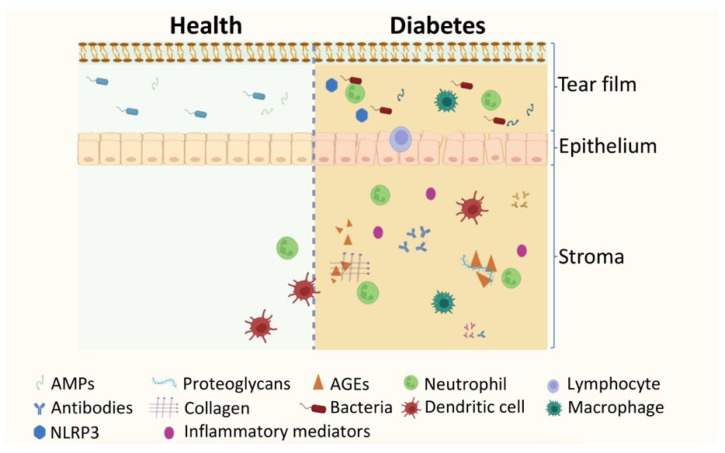
Schematic comparison of the homeostatic and diabetic immune environment of the ocular surface. In a healthy ocular surface, a variety of antimicrobial peptides (AMPs) and commensal bacteria are present in the tear film, providing protection and immunomodulation. The corneal stroma contains immune cells, such as neutrophils, macrophages, and dendritic cells, that contribute to maintaining the ocular surface´s homeostasis. However, in diabetes, homeostasis is disrupted. The production of advanced glycation end-products (AGEs) in diabetes triggers their deposition along the ocular surface, leading to the activation of the immune system and increased release of immunomodulators. This results in the release of inflammatory mediators, a marked inflammatory reaction, inflammasome activation, and changes in the microbiome. Consequently, the tear film’s quality and quantity are altered, leading to instability, hyperosmolarity, and impaired functions. The corneal epithelium undergoes abnormalities, such as erosion, increased permeability, and fragility, and collagen fibril bundles with different thicknesses and variable spacing are formed in the corneal stroma. These changes and abnormalities in proteoglycans lead to increased stromal thickness. This schematic illustration was created using BioRender.com, accessed on 4 May 2023.

During the initial stages of the normal wound healing process, there is an increase of the first circulating immune cells recruited to the injury site, neutrophils, which play a crucial role in clearing away cellular debris by phagocytosis, and they secrete antimicrobial peptides to fight off pathogens (Figure 3). However, these inflammatory cells can also produce signals that induce the recruitment and activation of additional neutrophils and other inflammatory cells like NK cells and macrophages. After accomplishing their role at the wound site, neutrophils undergo apoptosis and are engulfed by macrophages, signaling the end of inflammation. However, impairment in neutrophil function and metabolism is found in diabetes [135]. In diabetic wounded corneas, an increase in neutrophil numbers at the site of injury and a decrease in the number of dendritic cells have been found, which may limit macrophages’ ability to properly eliminate dying neutrophils [136,137]. The disturbance of epineuroimmune interactions may result in delayed wound healing and impaired sensory nerve regeneration in the cornea. Detailed defects in the epineuroimmune function unit in response to wounding in diabetes have been reviewed recently [137].

In another study, the ocular surface changes were shown to be associated with increased oxidative stress and decreased expression levels of silent information regulator 1 (SIRT1), FOXO3, and a member of the SOD family MnSOD [132]. SIRT1 downregulation has been found in mouse corneas from Ins2^Akita/+^ mice [138]. Findings in animal models and humans suggest that upregulation/activation of SIRT1 can suppress sensory neuronal degeneration pathways that lead to distal axonopathy [139].

Diabetes is known to be associated with inflammasome activation [140]. Inflammasomes are cytosolic receptors that recognize pattern structures on pathogens (pathogen-associate molecular patterns; PAMPs) or damaged tissues (danger-associated molecular patterns; DAMPs) [141]. NLRP3 (NOD-, LRR- and pyrin domain-containing protein 3) inflammasome is a member of the NOD-like receptor family that includes a sensor, the adaptor apoptosis speck-like protein (ASC), and the cysteine protease procaspase-1 [141]. The NLRP3 inflammasome, if activated, can trigger caspase-1 to convert pro-IL1β and pro-IL-8 to their active forms [142]. Increasing evidence suggests that NLRP3 inflammasome activation is implicated in diabetes and its complications, such as DR [143,144,145]. Recently, a diabetic mice model has shown ocular damage, such as impaired corneal epithelial integrity, decreased tear production, and decreased corneal sensitivity. Levels of reactive oxygen species (ROS) and expression of NLRP3, IL-1β, and caspase-1 have been found to increase in diabetic cornea and conjunctiva. These data suggest the contribution of ROS/NLRP3/caspase-1/IL-1β pathway activation in ocular surface damage in diabetes [146]. Other studies carried out on type 1 diabetes established Nlrp3 knockout (KO) C57BL/6 mice reinforced the contributions of NLRP3-inflammasome-mediated chronic inflammation and pyroptosis to diabetic keratopathy (DK) pathogenesis [140]. NLRP3 inflammasome was shown to be essential for corneal wound healing and nerve regeneration under physiological settings. However, in diabetic conditions, its sustained activation resulted in delayed corneal wound healing and poor nerve regeneration [140]. The build-up of advanced glycation end-products (AGEs) triggers an overactive NLRP3 inflammasome response by producing reactive oxygen species (ROS). Genetic and pharmacological blocking of AGEs/ROS/NLRP3 inflammasome improved corneal wound healing and nerve regeneration. Although these data suggest the pathogenic role of AGEs/ROS/NLRP3 inflammasome in the development of DK, this study did not assess the impact of normal and hyperactivation on epithelial and neural functions [140].

Given the close interplay between microbiota homeostasis and the host immune system, it is not surprising that the bacterial profile of the conjunctiva is changed in diabetes (Figure 3). Different bacterial flora has been described in diabetic and healthy rats, with emerged *Enterococcus casseliflavus*, *Enterococcus cecorum*, *Enterococcus faecalis*, *Enterococcus saccharolyticus*, *Kocuria kristinae*, *Enterobacter aerogenes*, and disappearance of *Klebsiella pneumoniae*, *Staphylococcus cohnii*, *Staphylococcus sciuri*, *Aerococcus viridans* in diabetic bulbar conjunctiva [147]. Gram-positive bacteria are the most frequently isolated microorganisms from the conjunctiva of both diabetic and healthy rats. However, it is worth noting that the two groups found three bacterial species in common. Additionally, seven new species emerged, while five disappeared in diabetes [147]. In healthy rats, the conjunctiva prominently harbors two species of *Staphylococcus*. However, in diabetic rats, these *Staphylococcus* species are replaced by *Enterococcus* species and other bacterial types. Enterococci are commonly found in both human and animal feces, and the changes in the bacterial composition may reflect alterations in the intestinal microbiota of diabetic rats [148]. The emerging bacteria in the conjunctiva of diabetic rats raises concerns about potential pathological infections in diabetic animals. For instance, *E. casseliflavus* has been reported to cause endogenous endophthalmitis in humans [149]. Similarly, *E. cecorum* and *K. kristinae* can cause human infections [150,151,152]. Thus, the emergence of these bacterial species in the conjunctiva of diabetic conditions could pose a risk of infection.

## 7. Mechanisms of DR Neurodegeneration and Vascular Lesions

DR is a progressive disease that develops in stages of increasing severity and is strongly influenced by the duration of diabetes [10], particularly in type 2 diabetes, which is the common form of diabetes. Within 20 years of being diagnosed with type 2 diabetes, nearly two-thirds of patients will exhibit some degree of retinopathy [1].

Understanding the natural history of DR is crucial for its classification. DR is a dynamic and progressive condition that undergoes distinct stages [14]. The first stage, known as preclinical retinopathy, manifests in microvasculopathy and neuropathy.

**Figure 4:**
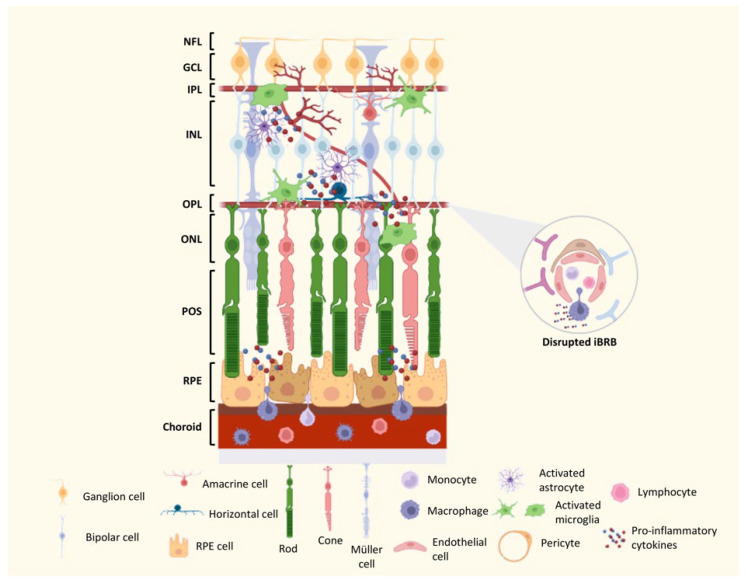
Schematic representation of the diabetic immune environment of the retina. During the progression of diabetes, the neurovascular unit (NVU) is dysregulated due to oxidative stress and inflammatory conditions. This triggers the activation of microglial cells, which retract their processes and migrate to the external layers of the retina, as well as the activation of Müller cells and astrocytes. Concurrently, the breakdown of the iBRB leads to the recruitment of peripheral immune cells, such as macrophages, and the subsequent release of pro-inflammatory cytokines into the neural retina. This leads to the degeneration of retinal neurons, including ganglion cells and photoreceptors. NFL—Nerve fiber layer; GCL—Ganglion cell layer; IPL—Inner plexiform layer; INL—Inner nuclear layer; OPL—Outer plexiform layer; ONL—Outer nuclear layer; POS—Photoreceptor outer segments; RPE cell—Retinal pigment epithelial cell. This figure was created using BioRender.com, accessed on 4 May 2023.

Over the years, our group and others have highlighted the critical and distinct roles that different retinal cells play in the pathogenesis of DR [153,154,155,156,157,158,159]. Neurodegenerative alterations in DR, including thinning of the retinal nerve fiber layer and the retina ganglion cell layer, are associated with functional visual deficits [160,161,162,163,164,165,166,167,168]. Furthermore, these neuronal structural changes not only overlap with the functional deficits, but they also appear to track the locations where future vascular lesions arise, implying that neurodegeneration contributes to visual dysfunction in preclinical DR and that there may be a link between neurodegeneration and vascular abnormalities in DR [15,169,170]. This is followed by the clinical stage, which involves morpho-structural and pathophysiological changes closely linked to progressive endothelial cell dysfunction. This dysfunction exacerbates the previous changes, ultimately leading to neovascularization (Figure 4) [171].

The classification and diagnosis of DR are based on the typical retinal microvascular lesions that become visible on fundoscopy during the clinical stage. According to the multicenter early treatment diabetic retinopathy study (ETDRS), DR is classified into NPDR and proliferative PDR based on these lesions [172].

A few microaneurysms characterize mild NPDR, while microaneurysms, intraretinal hemorrhages, or venous beading characterize moderate NPDR [173]. As the disease progresses, retinopathy may manifest with retinal hard exudates (lipid deposits in the retina from leakage lipoproteins), cotton wool spots (small localized infarctions in the nerve fiber layer of the retina) and intraretinal microvascular abnormalities (dilated capillary channels in areas of retinal ischemia). In the proliferative stage, new retinal blood vessels develop due to the upregulation of angiogenic factors in response to oxygen deprivation [174,175]. Additionally, fibrous tissue may develop at the optic disc or near venules in other parts of the retina. These new retinal blood vessels can bleed, leading to preretinal and vitreous hemorrhage, and the fibrovascular tissue can cause traction on the macula, resulting in loss of vision [14,176]. Diabetic macular edema, characterized by retinal thickening due to leaky blood vessels, represents the most common cause of vision loss in patients with diabetes and can develop at all stages of retinopathy, although it is more prevalent during later phases [14,173,177].

The exact pathophysiology of DR remains poorly understood, as the disease presents dynamic changes at different stages of its natural history and can vary from person to person [14]. However, sustained hyperglycemia-induced damage is believed to play a significant role in the development of DR, leading to various adaptive abnormalities in the diabetic retina [178]. These changes encompass biochemical, physiological, rheological, hormonal, and other alterations, which affect pathways and factors critical for the interaction, communication, and regulation of retinal cells. Notably, while endothelial cells are particularly susceptible to these changes, functional and morphological abnormalities can be found in various retinal cell types even before clinical symptoms and formal diagnosis of DR [179,180,181].

The pathogenesis of DR is complex, and several biochemical pathways have been implicated [173]. However, it is widely believed that dysregulation of the NVU plays a significant role in the development of the disease, although the exact mechanisms are still not fully understood. Hypoxia, inflammation, and oxidative stress have been identified as key mechanisms implicated in the onset and progression of DR [182].

Chronic hyperglycemia can increase the levels of various chemokines, including monocytic chemotactic protein-1 (MCP-1), CCL2, and CCL5, and proinflammatory cytokines, such as TNF-α, IL-1β, and IL-6. These pro-inflammatory mediators stimulate the secretion of intercellular adhesion molecule 1 (ICAM-1) and vascular cell adhesion molecule 1 (VCAM-1) by endothelial cells. These cell adhesion molecules play a key role in attracting circulating immune cells, such as leukocytes and monocytes, promoting adhesion molecule-mediated leukocyte–endothelial interaction, also known as leukocytosis [183]. Leukocytosis, a crucial event in the early stages of DR, can lead to the infiltration of inflammatory cells, resulting in more inflammation and damage to retinal tissue due to BRB disruption and loss of pericytes and endothelial cells (Figure 4) [184,185]. Moreover, it has been shown that the activation of NLRP3 inflammasome in the diabetic retina elicits a prolonged inflammatory response and BRB injury in an experimental model of diabetes [186].

The retina is susceptible to changes in oxygen levels due to its high content of polyunsaturated fatty acids (PUFAs) and its need to generate energy (ATP) through the mitochondrial electron transport chain (ETC) in the inner membranes, which consumes large amounts of glucose and oxygen [172,179,182]. During this process, reactive oxygen species (ROS) are generated as electrons leave the ETC and react with molecular oxygen [173,180,182]. The photoreceptors, which are the main source of superoxide and reactive oxygen in the retina, are particularly vulnerable to changes in homeostasis due to their limited capacity for mitochondrial reserve and, therefore, contribute significantly to oxidative stress. In response to the overproduction of ROS, the retina attempts to compensate by reducing its metabolic activity. However, in the case of diabetes, this compensatory mechanism fails, leading to damage to mitochondrial membrane lipids/proteins and mitochondrial DNA (mtDNA), resulting in inefficient ATP production and increased ROS production, which initiates the pathogenesis of DR [177,180,187].

When intracellular antioxidant enzymes in retinal cells are unable to remove ROS and other free radicals effectively, excessive ROS enters the cell nucleus, causing DNA strand breaks and triggering enzymes such as poly (ADP-ribose) polymerases (PARP) and poly ADP-ribose glycohydrolases (PARGs) [118,177,182,187,188,189]. This leads to reactive gliosis in Müller cells and astrocytes, as well as transcription of NF-κB. Once activated, NF-κB translates into the nucleus and promotes the expression of pro-inflammatory cytokines such as IL-1β, IL-6, IL-8, interferon, and TNFα. Additionally, the PI3K/Akt/mTOR pathway mediates the secretion of inflammatory cytokines in response to ROS induced by hyperglycemia. Furthermore, these pro-inflammatory molecules contribute to Müller cells induced-inflammation by stimulating the cluster of differentiation (CD) 40 and indirectly promoting microglial inflammation by releasing adenosine triphosphate. This inflammation state results in the up-regulation of ICAM-1, VCAM-1, MCP-1, and cyclooxygenase-2 (COX-2) [118,189]. COX-2 increases the synthesis of prostaglandins, which stabilize hypoxia-induced factor-1 (HIF-1), leading to vascular endothelial growth factor (VEGF) expression and NF-κB activation for COX-2 expression.

The exact mechanisms underlying microglia activation in DR are not fully understood. However, increased production of AGEs and ROS and release of DAMPs, along with Müller cells activation, can contribute to microglia activation. Moreover, it is known that, in healthy conditions, retinal neurons control microglia activation through CD200-CD200R and CXCL1-CXCR1 pathways. However, in DR, when there is neuronal dysfunction, these pathways become compromised, activating microglial cells [190]. ERK phosphorylation, triggered by ROS, is also involved in microglia activation [191]. Once activated, the microglia retract their processes, increase their proliferation and migration and release proinflammatory cytokines, such as IL-1β, TNF-α, and IL-6, and chemokines, such as MCP-1, ROS, and VEGF. Along with this, the phagocytic activity of microglia also increases [192]. Activated microglia can also produce neurotoxic factors, such as glutamate, caspase-3, and MMPs, promoting neurodegeneration [193].

When released into the cytosol, mitochondrial ROS and oxidized mtDNA are recognized as DAMPs by cytosolic PRRs, such as TLR4 and TLR9 receptors. This recognition, and pro-inflammatory responses, trigger various cell death processes, such as pyroptosis, apoptosis, and autophagy. The contribution of mitochondria to oxidative stress in diabetes is well-established [178]. According to the unified theory of hyperglycemia-induced endothelial cell damage proposed by Brownlee, ROS overproduction is the common upstream event that stimulates several biochemical pathways implicated in the pathogenesis of DR. These pathways include the polyol (sorbitol) pathway, formation of AGEs intracellularly, expression of receptors of AGEs (RAGE) and ligands [176,194], activation of the protein kinase C (PKC) pathway, and increased activity of the hexosamine pathway [14,118,176,189,195,196].

Oxidative stress, in conjunction with chronic hyperglycemia and low-grade inflammation, plays critical roles in the onset and progression of DR, as they collectively contribute to the breakdown of the BRB and neovascularization [173]. The damage to retinal cells, the neovascularization process, and the overall pathogenesis of DR result from oxidative stress directly or indirectly stimulating the release of pro-inflammatory cytokines, VEGF-α, and nitric oxide (NO) [197,198]. In summary, hyperglycemia triggers a cascade of events leading to the development of DR, involving microvascular dysfunction characterized by increased production of pro-inflammatory and pro-oxidant mediators or endogenous signaling pathways [14,171,199,200,201].

Recent evidence highlighted the complement system’s key role as an important factor in DR progression. Complement system activation may be implicated in the etiology of the microvascular disease by inducing systemic inflammation and contributing to microvascular endothelial cell dysfunction [202]. Increased plasma complement C3 has been associated with an increased risk of DR [202]. Moreover, increased concentrations of C5a detected in the vitreous of diabetic patients with PDR were significantly correlated with the concentrations of VEGF [202]. C5b-9, the terminal product of the complement activation, was found in the wall of retinal vessels of human eye donors with 9 ± 3 years of type 2 diabetes [203]. CFH expression was also shown to be induced by IL-27 in mouse retinal cells [204]. IL-27 signaling in T cells changes the balance of regulatory T cells (Tregs) and T helper1 (Th1) cells, and effector cells (CD8 T cells) and is critical for T1D development as well as lacrimal gland inflammation in nonobese mice [205]. The levels of this cytokine are increased in aqueous humor in patients with DR compared to healthy control individuals [206]. However, further research is needed to understand how innate immune cells and the complement system are regulated in DR.

## 8. The Contribution of the Immune Response to DR in Preclinical Models

Like early DR in humans, recent data have shown that in a preclinical rat model, thinning of the inner layers of the retina, indicating neuronal dysfunction, may occur prior to vascular and inflammatory changes [207]. This finding parallels the observations made in human cases of DR [208]. Several studies have demonstrated that the activation of the immune-competent microglia may be implicated in neurodegeneration in DR. Their activation occurs prior to neuronal thinning, and microglia possess the ability to eliminate impaired neurons by phagocytosis [209,210]. Recently, a significantly higher number of activated Iba-1+ microglial cells were described in the retina of the type 1 diabetes animal model (Akita mice) compared to aged-matched wild-type mice [211]. Interestingly, the authors found that the diabetic globe and retina retain bacteria. The diabetic retina presented a distinct pattern of microbes in the retina and increased levels of the microbial peptide PGN and TLR2, which can contribute to the disruption of the BRB, driving the loss of the immune-privilege state of the ocular system [212]. The NLRP3 inflammasome activation in diabetes and its potential relationship with the pathogenesis of DR has been recently reviewed [213].

The top 10 genera identified in the globes were Akkermansia, Bacteroides, Bifidobacterium, Clostridium, Corynebacterium, Faecalibacterium, Lactobacillus, Propionibacterium, Pseudomonas, and Staphylococcus. Decreased abundance of Corynebacterium and Pseudomonas and an increased abundance of Lactobacillus, Staphylococcus, Enterococcus, and Bacillus were found in the retina of Akita mice [211].

Lactobacillus is known to generate lactic acid as its end product through a process known as homofermentative metabolism. Retinal ganglion cells and Muller cells express the lactic acid receptor, the hydroxycarboxylic acid receptor (HCAR1/GPR81), that, when activated by the lactic acid, can lead to a variety of responses, including changes in gene expression and alterations in cell metabolism [214]. Lactic acid has also been found to modulate VEGF expression and produce numerous angiogenic factors, including Norrin, contributing to angiogenesis. Tail vein-injected Staphylococcus aureus (mimicking a systemic inflammatory state) can increase BRB permeability and promote endogenous bacterial endophthalmitis (intraocular infection triggered by a bloodstream infection) in both control and diabetic mice, but its incidence is higher in the later [215]. Bacillus, which is one of the leading causes of endogenous endophthalmitis and is also able to compromise the BRB, was also found to be elevated in diabetic mouse retinas. Furthermore, overpopulated Enterococci have previously been correlated to gut-barrier disruption and systemic inflammation through the activation of TLRs [216]. It can have a similar role in BRB dysfunction. Altogether, these findings suggest that the presence of potentially pathogenic microbes may result in the production of pro-inflammatory cytokines by retinal cells, contributing to an inflammatory retinal environmental and DR pathogenesis. Although it is still not well understood how pathogens are recognized, and inflammation is initiated in the retina, the retinal resident immune cells, microglia, and Müller cells that express TLRs seem to have a key role in those processes. They can elicit innate immune responses to particular pathogens’ invasion in the vitreous cavity [217]. TLRs, which are well-recognized PRRs, upon recognition of PAMPs, can induce inflammatory mediators and production of AMPs [218]. Müller cells are activated in response to bacterial challenge, showing increased levels of TLR2 and exhibiting bactericidal properties, generating a variety of AMPs, including LL-37 (cathelicidin-related AMP (CRAMP) for mouse), according to in vivo and in vitro studies [219,220]. Moreover, another study reported the critical role of cathelicidins in modulating the response of neutrophils and the release of inflammatory mediators [217]. However, to the best of our knowledge, the impact of diabetes on CRAMP levels in retinal cells is not known.

## 9. Conclusions and Perspectives

Over the last decade, significant progress has been made in understanding the molecular and cellular mechanisms underlying ocular complications of diabetes. We have highlighted the advances of research in this topic by outlining the harmful effects of chronic hyperglycemia on the status of the ocular immune-privileged sites in the eye crucial for ocular homeostasis maintenance and organ integrity: ocular surface and retina. Both structures have different anatomical barriers: corneal and conjunctival epithelium covers the ocular surface and act as a barrier between the ocular surface and the interior of the eyeball; tear film also contributes to a refractive and barrier function against the hostile environment; inner and outer BRB are physiological barriers that selectively regulate substances flux into and out of the retina. The ocular tissues, including the cornea and retina, comprise a complex structure of innate immune cells (resident macrophages and dendritic cells) that secrete a variety of mediators that support the immune privilege. Understanding the pathophysiology of DR requires understanding how the ocular microenvironment contributes to modifying the immune system and breaches of privilege.

There is no doubt that rodent models of diabetes provide great insight into the mechanisms underlying ocular complications of diabetes and contribute to clarifying how these mechanisms may be interconnected. However, no single rodent accurately mirrors human diabetic eye diseases. Overall, while the ocular immune system in rodents and humans is similar in many ways, some important differences can affect how the immune response is localized and regulated in the eye. These differences must be considered when studying the ocular immune response in animal models and translating the findings to humans. Regarding the immune cells in the eye, rodents and humans have similar types of immune cells, such as T cells, B cells, macrophages, and dendritic cells, which play critical roles in protecting the eye from infection and inflammation. However, the relative proportions of these cells may differ between rodents and humans, which can affect the overall immune response. Additionally, rodents’ lymphatic vessels and lymph nodes differ from those in humans, which can affect how immune cells migrate in and out of the eye.

The study of the crosstalk between immune cells at the ocular surface and retina and understanding the impact of diabetes is an active field of research. There is still much that is not known about the mediators involved. However, the relevance of this research is significant as it has the potential to improve our understanding of the development and progression of diabetic eye diseases such as DK and DR, which can lead to significant vision loss if not treated properly. The lack of knowledge in this field represents an opportunity for further research and discovery, which can help identify new targets for treating these diseases. For example, understanding the specific cytokines and signaling pathways involved in the crosstalk between immune cells at the ocular surface and retina could lead to the development of new drugs that can target these molecules and reduce inflammation in the eye. Additionally, research in this field can also help identify new diagnostic and prognostic markers for ocular diseases, aiding in early detection and treatment.

Overall, studying the crosstalk between immune cells at the ocular surface and retina is important as it can help improve our understanding of ocular diseases and lead to the development of new treatments that can improve the quality of life for people affected by these conditions.

## Data Availability

Not applicable.
